# Serum concentrations of lipids, ketones and acylcarnitines during the postprandial and fasting state: the Postprandial Metabolism (PoMet) study in healthy young adults

**DOI:** 10.1017/S0007114524001934

**Published:** 2024-10-14

**Authors:** Åslaug Matre Anfinsen, Vilde Haugen Myklebust, Christina Osland Johannesen, Jacob Juel Christensen, Johnny Laupsa-Borge, Jutta Dierkes, Ottar Nygård, Adrian McCann, Hanne Rosendahl-Riise, Vegard Lysne

**Affiliations:** 1 Mohn Nutrition Research Laboratory, Centre for Nutrition, Department of Clinical Science, University of Bergen, Bergen, Norway; 2 Department of Nutrition, Institute of Basic Medical Sciences, University of Oslo, Oslo, Norway; 3 Bevital AS, Bergen, Norway; 4 Mohn Nutrition Research Laboratory, Centre for Nutrition, Department of Clinical Medicine, University of Bergen, Bergen, Norway; 5 Laboratory Medicine and Pathology, Haukeland University Hospital, Bergen, Norway; 6 Department of Heart Disease, Haukeland University Hospital, Bergen, Norway

**Keywords:** Postprandial response, Fasting, Metabolism, Metabolites, Metabolomics, Biomarkers, Epidemiology, Carnitine, Acylcarnitines

## Abstract

To improve the interpretation and utilisation of blood lipids, ketones and acylcarnitine concentrations as biomarkers in clinical assessments, more information is needed on their dynamic alterations in response to dietary intake and fasting. The aim of this intervention study was to characterise the changes in serum lipid, ketone and acylcarnitine concentrations 24 h after a standardised breakfast meal. Thirty-four healthy subjects (eighteen males and sixteen females) aged 20–30 years were served a breakfast meal (∼500 kcal, 36 E% fat, 46 E% carbohydrates, 16 E% protein, 2E% fibre), after which they consumed only water for 24 h. Blood samples were drawn before and at thirteen standardised timepoints after the meal. Metabolite concentrations were plotted as a function of time since the completion of the breakfast meal. Results demonstrated that concentrations of HDL-cholesterol and LDL-cholesterol decreased until ∼2 h (–4 % for both), while TAG concentrations peaked at 3 h (+27 %). Acetoacetate and *β*-hydroxybutyrate were highest 24 h after the meal (+433 and +633 %, respectively). Acetylcarnitine, butyrylcarnitine, hexanoylcarnitine, octanoylcarnitine, decanoylcarnitine and dodecanoylcarnitine reached the lowest values at 60 min (decreases ranging from –47 to –70 %), before increasing and peaking at 24 h after the meal (increases ranging from +86 to +120 %). Our findings suggest that distinguishing between fasting and non-fasting blood samples falls short of capturing the dynamics in lipid, ketone, carnitine and acylcarnitine concentrations. To enhance the utility of serum acylcarnitine analyses, we strongly recommend accounting for the specific time since the last meal at the time of blood sampling.

Blood biomarkers are often measured in blood samples taken in fasting samples at least 6–8 h post dietary intake. However, individuals in Western societies typically spend a substantial portion of their awake hours in the postprandial state, the hours following a meal, and only enter a fasting state during an overnight sleep^(1)^. Thus, fasting samples might not fully capture the true aetiological exposure of a biomarker. Moreover, it is common in clinical care and in research settings to distinguish between fasting and non-fasting blood samples. However, the transition between the non-fasting and the fasting state is a gradual process, and there is reason to believe that the concentrations of biomarkers may change gradually during this transition.

The blood lipids TAG, LDL-cholesterol and HDL-cholesterol are well-established risk factors for CVD. It has been shown that blood lipid concentrations change as a response to dietary intake. For instance, Langsted and colleagues^(2)^ reported that TAG concentrations were increased with +0·3 mmol/l at 1–4 h, while LDL-cholesterol concentrations were decreased with −0·2 mmol/l the first 2 h after dietary intake. As the lipid concentrations may change after dietary intake, the measurement of blood lipids has traditionally entailed samples taken at a minimum of 6–8 h post dietary intake^(3)^. However, notably, evidence suggests that a non-fasting lipid profile may be superior to fasting for predicting cardiovascular risk, leading to updated clinical guidelines allowing for non-fasting lipid testing for most evaluations^(4,5)^.

During prolonged fasting, when glucose is not readily available, ketone bodies become an important alternative source of energy. Ketone bodies are synthesised mainly from acetyl-CoA generated by the *β*-oxidation of fatty acids^(6)^. In order for these fatty acids to undergo *β*-oxidation, acylcarnitines are needed to transport the fatty acids across the mitochondrial membrane, providing a link between the acylcarnitines and the ketone bodies. In the clinic, the ketone bodies are established biomarkers for diabetic ketoacidosis; however, they have recently been explored as biomarkers for other conditions, such as heart failure^(7)^. Moreover, a growing interest has emerged in utilising acylcarnitines as potential biomarkers for a spectrum of diseases, such as CVD^(8,9)^, diabetes^(10,11)^, chronic fatigue syndrome^(12)^ and depression^(13)^. As of today, acylcarnitines are measured in the newborn screening for inborn errors of fatty acid oxidation, such as carnitine-acylcarnitine translocase deficiency or carnitine transporter defect, among others. However, beyond the newborn screening, acylcarnitines are not routinely measured in the clinic^(14)^. To establish the credibility of ketones and acylcarnitines as reliable biomarkers, a crucial prerequisite is to determine their variations in serum levels in response to dietary intake and fasting^(15)^.

By unravelling how lipids, ketones and acylcarnitines change as a response to dietary intake, we can improve the interpretation and utilisation of these metabolites as biomarkers in clinical assessments and in epidemiological research. Moreover, given that metabolite concentrations can differ by sex^(16–19)^, it is likely that concentration responses might also be sex-dependent. Hence, investigating sex-specific responses to dietary intake is warranted.

We have previously reported data on how amino acids, one-carbon metabolites and B-vitamin biomarkers change 24 h after intake of a standardised meal in healthy, young adults^(20)^. In the present study, using data from the same controlled trial, we aimed to characterise the overall and sex-specific changes in serum concentrations of lipids, ketones and acylcarnitines.

## Methods

### Recruitment and pre-screening

Detailed information on the recruitment of participants in the interventional Postprandial Metabolism (PoMet) study has been described previously^(20)^. The inclusion criteria for participation were (1) aged 20–30 years (birth years 1991–2001) and (2) self-reported BMI of 22–27 kg/m^2^ at phone screening. Subjects were excluded if they (1) had experienced acute or chronic disease such as diabetes, thyroid diseases, cancer, CVD or inflammatory bowel disease during the last 3 years, (2) had celiac disease or other food allergies interfering with the standardised breakfast meal, (3) used any prescription medications except for contraceptives; (4) smoked or used other nicotine-containing products such as ‘snuff’ regularly, (5) had been pregnant or breastfed the last three months before study visit and (6) had experienced weight change > 5 % during the last 3 months before the study visit. Individuals who were interested in participating in the study were contacted and pre-screened over the phone, and individuals eligible for inclusion were invited to the main screening at the study visit.

### Instructions prior to the study visit

To standardise physiological and metabolic conditions prior to the study visit, all individuals were instructed to (1) not use dietary supplements the last 7 d before the visit, (2) abstain from smoking and use of nicotine-containing products such as ‘snuff’ the last 7 d before the visit, (3) abstain from alcohol and avoid any strenuous activity the last 24 h before the visit, (4) consume an evening meal consisting of three slices of bread with cheese and jam and a glass of juice at 08.00 the evening before the study visit, (5) not consume anything other than water after the evening meal before the study visit and (6) drink enough water and to stay hydrated to facilitate blood sampling and the insertion of a venous catheter.

### Main screening and study visits

The study visits were conducted at the Research Unit for Health Surveys at the University of Bergen, Norway. At attendance 07.30, all individuals were screened and had their height and body weight measured to calculate their BMI for screening purposes. The main screening has been described in more detail previously^(20)^. Participants who fulfilled all inclusion and exclusion criteria and provided informed consent were included in the study.

After the main screening, a venous blood sample was drawn, and thereafter, a standardised breakfast meal was served. All participants were instructed to consume the breakfast in precisely 15 min, and the minute the last bite was consumed was set to timepoint zero. After the breakfast meal was consumed, the participants were instructed not to consume anything other than water (no chewing gum, sparkling water, diet soda, etc.) for the next 24 h. Venous blood samples were drawn at thirteen standardised timepoints after the meal: 15 min, 30 min, 45 min, 60 min, 90 min, 2 h, 3 h and 4 h after the meal, and then every other hour until 12 h after the meal. After the 10 h blood sample, body composition was analysed using a BodPod (COSMED, version 5·4·6). Detailed information about the body composition analysis has been described previously^(20)^. After the 12 h blood sample, the participants left the study centre overnight and came back the next morning for the last blood sample taken 24 h after the breakfast meal.

### Blood sampling and laboratory analyses

All blood samples were drawn following standardised procedures with participants sitting in an upright position. At each timepoint, a total of 11·5 ml of blood was drawn and distributed into serum tubes (8·5 ml, BD Vacutainer^®^ SST^TM^ II *Advance*; Beckton, Dickinson, and Company) and EDTA tubes (3 ml, Vacuette^®^ K2EDTA). At baseline and at the 24 h timepoint, an additional 6 ml and 3 ml EDTA blood was collected, respectively, for measurement of haematology and routine clinical biomarkers (aminotransferases, creatinine, C-reactive protein, erythrocytes, *γ*-glutamyltransferase, Hb, HbA1c, mean corpuscular Hb, mean corpuscular volume, mean platelet volume, thrombocytes, thyroid stimulating hormone and 25-hydroxyvitamin D)^(20)^. The first twelve blood samples were drawn through a venous catheter in the elbow cavity. After the 10 h blood sample, the venous catheter was removed to facilitate the measurement of body composition. Consequently, the last two blood samples (12 and 24 h after the meal) were drawn as normal venous blood samples.

After the blood sampling, the serum tubes were stored at room temperature for 30–60 min and then centrifuged at 2200 *g* for 10 min at 20°C. Three aliquots of serum per timepoint were temporarily stored in a freezer at –20°C and transferred to –80°C at the end of the day. Additionally, one aliquot of serum from each timepoint was stored in the refrigerator at +4°C and transported to the laboratory daily. The lipids were analysed at the Department of Medical Biochemistry and Pharmacology at Haukeland University Hospital, Bergen, Norway (certified NS−EN ISO 15189:2012) using photometry. Serum concentrations of the ketone bodies, *β*-hydroxybutyrate (*β*HB) and acetoacetate (AcAc), were measured by adding ion pairs for the analytes and isotope-labelled internal standards to an existing gas chromatography−tandem mass spectrometry (GC−MS/MS) assay^(21)^. Within- and between-day CV for *β*HB and AcAc ranged from 2 to 4 %. Serum concentrations of free carnitine and the short- and medium-chain acylcarnitines were analysed by adding ion pairs for the analytes and isotope-labelled internal standards to an existing liquid chromatography−tandem mass spectrometry (LC−MS/MS) assay^(22)^. Within- and between-day CV for carnitine and the acylcarnitines ranged from 4 to 8 %. Both the ketones and carnitines were measured at Bevital AS, Bergen, Norway.

### The breakfast meal

The same standardized breakfast meal was provided to all participants. The meal contained ∼500 kcal (36 E% fat, 46 E% carbohydrates, 16 E% protein, 2E% fibre) and consisted of 90 g whole-grain wheat bread with 15 g butter, 20 g strawberry jam, 40 g low-fat cheese (16 % fat), 36 g cucumber and a glass (200 ml) of orange juice. Detailed information about the nutrient composition of the breakfast meal has been described previously^(20)^. The breakfast meal was composed to mimic a normal Norwegian breakfast and provided 20–24 % of the estimated daily energy needs, which are estimated to be 2600 kcal and 2150 kcal per d for inactive males and females, respectively^(23)^.

### Statistical analyses

Detailed information about the sample size calculation and statistical analyses has been described previously^(20)^. All statistical analyses were performed using R v.4.1.3 (R Foundation for Statistical Computing, −https://www.r−project.org/), including the *tidyverse* packages (https://www.tidyverse.org) and the *irrICC* package v.1·0.

All metabolite concentrations were log-transformed before statistical analysis and described using the back-transformed *gMean* and *gSD* as recommended^(24,25)^. Inferential statistics are accompanied by 95 % geometric compatibility (confidence) intervals (*gCI*) as a measure of uncertainty, calculated using the geometric standard error (*gSE*) and formulas 1–3:
(1)

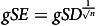



(2)

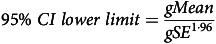



(3)

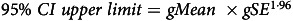




The main objective is presented visually, by plotting the raw metabolite concentrations as a function of time, with the mean time course indicated by superimposing the geometric mean concentrations (95 % *gCI*) on top of the individual data. Relative changes in metabolite concentrations were calculated for each individual, with each pre-breakfast blood sample utilised as an individual reference value. These individual percentage changes were subsequently combined to calculate the *gMean* percentage change across the study cohort. To evaluate the degree to which the different biomarkers are affected during the postprandial period, the within-person reproducibility was quantified by calculating intraclass correlation coefficients on log-transformed data. The intraclass correlation coefficients were calculated on the basis of a two-way random-effects model for absolute agreement, using the *irrlCC* package and the function *icc2.nointer.fn()* ^(26)^. Metabolite concentrations below the limit of detection were handled by replacing the missing values with the limit of detection divided by 2.

#### Sample size calculation

The sample size calculation was performed using an accuracy-in-parameter-estimation approach, as recommended when the main purpose is to accurately estimate the parameters of interest^(20,21)^. For the main analysis, we aimed to achieve a multiplicative margin of error (*gSE* 1·96) < 1·10, corresponding to a geometric standard error (*gSE*) < 1·05, for at least 80 % of the measurements. Using freely available data on 132 metabolites across 56 timepoints (7392 estimates) across different metabolic challenges from the HuMet study^(27)^ (available from http://metabolomics.helmholtz-muenchen.de/humet/), the observed median (80th percentile) *gSD* was 1·24 (1·32). Rearranging equation 1 above, and solving for *n* with a *gSD* = 1·32, we needed a sample size of 32 to achieve the desired precision level. We expected a dropout of up to 10 % due to adverse events following fasting blood sampling or difficulties drawing blood from a venous catheter. Therefore, to achieve our goal of collecting complete data for thirty-two participants, we aimed to recruit a total of thirty-six participants (eighteen males and eighteen females).

## Results

### Participant characteristics

Detailed participant characteristics have been described previously^(20)^. A flow chart depicting the inclusion of participants is illustrated in online Supplementary Fig. 1. A total of eighteen males and sixteen females were included in the analyses. The age ranged from 20 to 30 years old (mean age 25 years), while the BMI ranged from 20·2 to 26·9 kg/m^2^ (mean BMI 23·5 kg/m^2^). Among the females, thirteen of sixteen participants reported to use some form of contraceptives. The male participants had, on average, slightly higher BMI than female participants.

### Metabolite concentrations in the hours after the meal

A complete overview of the relative changes in metabolite concentrations is provided in Table 1 (total cohort) and Table 2 (for males and females separately). Additionally, an overview of geometric and arithmetic mean and range values in baseline concentrations is provided in online Supplementary Table 1. Absolute concentrations at all timepoints are presented in online Supplementary Table 2 (total cohort) and online Supplementary Table 3 (males and females separately). Figures illustrating the relative change in biomarker and metabolite concentrations for the total population can be found in online Supplementary Figs. 2–4.

**Table 1.** Relative changes in serum metabolite concentrations (% changes from reference values) after consumption of a standardised meal in healthy subjects in the Postprandial Metabolism study

	Time after meal														
	Ref*	15 min	30 min	45 min	60 min	90 min	2 h	3 h	4 h	6 h	8 h	10 h	12 h	24 h	ICC
*n*	34	34	34	34	34	34	34	33	33	33	33	33	33	33	−
Ketones															
Acetoacetate	37·4 µmol/l	–35·5	–28·4	–27·3	–22·0	–29·8	–24·6	–18·3	8·37	77·0	110	191	269	433	0·33
*ß*-hydroxybutyrate	62·6 µmol/l	–45·9	–48·3	–53·2	–56·2	–63·1	–60·1	–42·2	3·71	123	196	334	442	633	0·24
Lipids															
HDL-cholesterol	1·55 mmol/l	–1·35	–3·49	–3·54	–3·30	–4·14	–3·90	–3·50	–3·08	–0·14	2·19	3·9	5·97	0·81	0·93
LDL-cholesterol	2·51 mmol/l	–1·77	–3·82	–3·90	–3·40	–3·85	–4·30	–2·77	–1·85	1·02	3·29	5·62	6·64	6·91	0·85
TAG	0·85 mmol/l	1·24	6·58	10·1	11·1	15·5	19·9	26·5	25·9	–1·46	–14·3	–16·6	–14·6	13·3	0·76
Carnitines															
Free carnitine	35·1 µmol/l	3·61	6·31	9·28	10·6	14·3	17·0	16·3	10·8	0·45	–3·38	–7·5	–9·95	1·62	0·52
Short-chain acylcarnitines															
Acetylcarnitine (C2)	8·45 µmol/l	–8·88	–17·6	–25·0	–31·5	–39·2	–40·3	–32·1	–11·7	14·1	22·8	27·9	35·1	63·1	0·45
Propionylcarnitine (C3)	0·32 µmol/l	0·16	2·22	6·18	10·2	15·5	19·2	13·2	–0·90	–19·6	–21·5	–25·3	–25·6	–7·53	0·62
Butyrylcarnitine (C4)	0·25 µmol/l	–5·33	–6·18	–3·62	–2·27	1·11	6·35	7·14	–2·17	–16·4	–19·3	–22·3	–17·7	16·6	0·76
Isovalerylcarnitine (iC5)	0·12 µmol/l	–9·45	0·22	4·93	11·8	11·6	14·6	14·1	0·10	–17·2	–15·9	–17·1	–18·7	6·30	0·60
Glutarylcarnitine (C5-DC)	0·07 µmol/l	–2·31	3·55	–3·20	–3·73	–1·83	–5·52	–8·14	–6·72	–8·42	–3·72	–5·19	–0·43	1·78	0·69
Medium-chain acylcarnitines															
Hexanoylcarnitine (C6)	0·03 µmol/l	–20·4	–33·7	–46·1	–46·9	–45·8	–40·3	–26·4	–16·9	–7·59	9·82	19·1	35·3	114	0·48
Octanoylcarnitine (C8)	0·14 µmol/l	–21·7	–46·6	–63·7	–69·8	–65·8	–60·5	–49·2	–30·8	–23·9	–17·1	–18·4	9·87	85·9	0·47
Decanoylcarnitine (C10)	0·30 µmol/l	–24·5	–45·9	–61·9	–66·1	–62·8	–56·1	–40·7	–29·9	–21·3	–10·7	–8·73	14·0	107	0·47
Dodecanoylcarnitine (C12)	0·07 µmol/l	–17·5	–36·8	–49·6	–57·5	–58·1	–51·6	–36·3	–24·6	–16·4	–4·62	0·71	21·4	120	0·47

ICC, intraclass correlation coefficient; DC, dicarboxyl.

* Reference values are given in geometric mean. The reference values were measured at 08.00, 12 h after a standard evening meal. The relative changes were calculated as ratio to baseline values and thereafter combined to calculate the geometric mean percentage change across the study cohort.

**Table 2.** The relative change in metabolite concentrations (% changes from reference values) after consumption of a standardised meal in male (*n* 18) and female (*n* 16) participants in the postprandial metabolism study

		Time after meal														
	Sex*	Ref^†^	15 min	30 min	45 min	60 min	90 min	2 h	3 h	4 h	6 h	8 h	10 h	12 h	24 h	ICC
*n*		34	34	34	34	34	34	34	33	33	33	33	33	33	33	−
Ketones																
Acetoacetate	M	40·9 µmol/l	–33·0	–25·6	–29·3	–24·5	–31·8	–30·0	–20·9	0·64	73·7	104	164	261	435	0·20
	F	33·8 µmol/l	–38·2	–31·4	–24·9	–19·0	–27·5	–18·1	–15·1	18·4	81·1	117	228	280	430	0·37
* ß*-hydroxybutyrate	M	63·8 µmol/l	–42·5	–48·1	–54·1	–57·6	–63·9	–61·6	–39·7	–1·04	107	187	288	407	652	0·13
	F	61·2 µmol/l	–49·6	–48·5	–52·1	–54·6	–62·2	–58·4	–45·0	9·72	143	207	395	488	612	0·30
Lipids																
HDL-cholesterol	M	1·45 mmol/l	–1·15	–4·22	–3·88	–3·86	–3·65	–3·79	–3·58	–3·01	–0·47	1·80	4·12	5·80	1·10	0·94
	F	1·66 mmol/l	–1·57	–2·67	–3·15	–2·67	–4·68	–4·01	–3·40	–3·15	0·26	2·66	3·63	6·18	0·46	0·87
LDL-cholesterol	M	2·58 mmol/l	–1·19	–3·24	–3·94	–3·00	–3·88	–3·48	–2·36	–0·92	1·29	2·74	5·65	7·09	8·51	0·97
	F	2·44 mmol/l	–2·42	–4·47	–3·85	–3·86	–3·82	–5·22	–3·27	–2·94	0·71	3·95	5·58	6·10	5·01	0·70
TAG	M	0·90 mmol/l	2·49	6·44	10·4	13·8	20·6	26·0	29·7	29·5	–3·14	–15·0	–18·2	–16·3	12·4	0·75
	F	0·81 mmol/l	–0·16	6·75	9·81	8·11	10·1	13·5	22·7	21·8	0·58	–13·5	–14·7	–12·5	14·3	0·78
Carnitines																
Free carnitine	M	38·3 µmol/l	3·39	6·07	8·39	10·7	12·2	15·4	13·7	8·76	0·01	–3·70	–6·97	–8·51	2·30	0·51
	F	31·8 µmol/l	3·86	6·59	10·3	10·5	16·7	18·7	19·5	13·3	0·98	–2·99	–8·12	–11·7	0·80	0·51
Short-chain acylcarnitines																
Acetylcarnitine (C2)	M	8·86 µmol/l	–6·41	–15·4	–22·5	–29·3	–38·1	–39·0	–28·9	–15·0	8·32	17·7	19·3	27·8	60·3	0·40
	F	8·01 µmol/l	–11·6	–20·1	–27·8	–34·0	–40·4	–41·7	–35·7	–7·60	21·5	29·2	38·9	44·6	66·4	0·45
Propionylcarnitine (C3)	M	0·36 µmol/l	2·00	4·08	6·61	11·0	16·3	18·2	10·6	–2·78	–20·1	–21·7	–25·9	–25·2	–9·97	0·53
	F	0·29 µmol/l	–1·88	0·17	5·69	9·29	14·7	20·4	16·3	1·39	–19·0	–21·3	–24·6	–26·1	–4·52	0·57
Butyrylcarnitine (C4)	M	0·28 µmol/l	–6·89	–7·93	–7·23	–6·03	–1·56	0·30	0·77	–9·23	–18·5	–20·4	–24·1	–16·4	19·9	0·79
	F	0·22 µmol/l	–3·56	–4·17	0·61	2·13	4·20	13·6	15·3	7·03	–13·9	–18·1	–20·0	–19·2	12·7	0·65
Isovalerylcarnitine (iC5)	M	0·13 µmol/l	–7·61	–0·55	4·41	10·3	12·7	14·6	9·06	–3·14	–19·9	–18·6	–16·3	–12·2	6·14	0·66
	F	0·11 µmol/l	–11·5	1·10	5·51	13·5	10·4	14·7	20·5	4·14	–14·0	–12·5	–18·1	–25·8	6·50	0·54
Glutarylcarnitine (C5-DC)	M	0·07 µmol/l	–1·35	4·89	–3·9	1·21	5·52	–4·47	–4·56	–7·21	–5·79	–1·61	1·28	–1·89	1·74	0·84
	F	0·06 µmol/l	–3·38	2·05	–2·41	–9·00	–9·49	–6·68	–12·3	–6·13	–11·5	–6·19	–12·4	1·34	1·83	0·59
Medium-chain acylcarnitines																
Hexanoylcarnitine (C6)	M	0·04 µmol/l	–18·8	–32·0	–43·2	–43·5	–44·6	–37·6	–25·8	–19·7	–8·27	7·73	10·9	26·1	113	0·26
	F	0·03 µmol/l	–22·3	–35·6	–49·2	–50·5	–47·2	–43·1	–27·1	–13·3	–6·77	12·4	29·8	47·3	115	0·49
Octanoylcarnitine (C8)	M	0·18 µmol/l	–19·7	–40·5	–61·3	–69·3	–62·8	–54·9	–43·0	–25·5	–22·3	–14·9	–18·3	6·26	86·7	0·30
	F	0·11 µmol/l	–23·9	–52·7	–66·1	–70·3	–68·9	–66·0	–55·7	–36·8	–25·8	–19·6	–18·4	14·4	85·0	0·44
Decanoylcarnitine (C10)	M	0·36 µmol/l	–23·5	–42·5	–58·1	–61·6	–56·7	–50·2	–32·8	–25·2	–19·1	–9·53	–8·57	4·39	112	0·31
	F	0·24 µmol/l	–25·6	–49·6	–65·8	–70·4	–68·6	–61·8	–48·9	–35·1	–23·9	–12·0	–8·91	26·7	103	0·46
Dodecanoylcarnitine (C12)	M	0·08 µmol/l	–12·2	–32·2	–46·5	–55·9	–50·7	–48·3	–27·9	–19·8	–17·0	–5·88	–7·05	12·3	112	0·28
	F	0·06 µmol/l	–23·1	–41·7	–52·9	–59·2	–65·2	–55·0	–45·1	–30·1	–15·5	–3·08	10·9	33·3	130	0·47

DC, dicarboxyl.

*Male/female. ^†^Reference values are given in geometric mean and were determined from the pre-breakfast blood draw. The relative changes were calculated as ratio to baseline values and thereafter combined to calculate the geometric mean percentage change across the study cohort.

#### Blood lipids

**Fig. 1.**
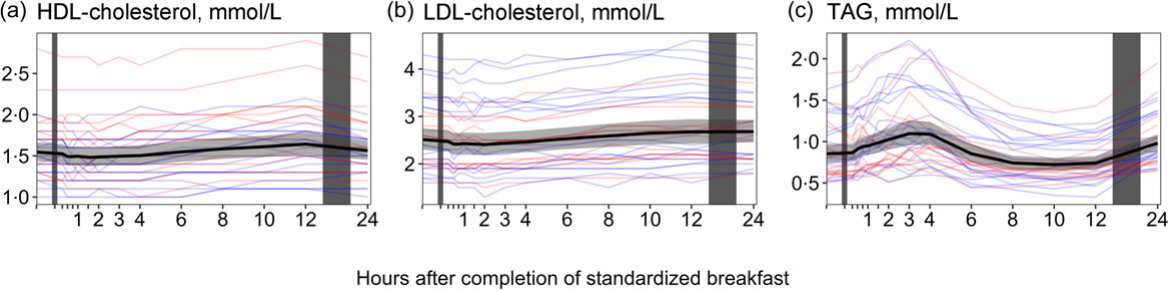
The concentrations of the blood lipids as a function of time since completion of the standardised breakfast meal in participants in the Postprandial Metabolism study (*n* 34). The solid black line represents the geometric mean, while the grey-shaded area represents the 95 % geometric CI. The blue and red lines represent the male and female participants, respectively. The leftmost vertical line indicates the time of the standardised breakfast meal, while the rightmost vertical line indicates time spent outside the study centre.

For HDL-cholesterol (Fig. 1(a), Table 1), we observed a slight decrease in concentrations during the initial hours following the meal, with a decrease of −0·06 mmol/l (−4·1 %) 90 min after the meal. However, the concentrations gradually increased and reached the highest values at 12 h, with an increase of +0·09 mmol/l (+6·0 %). For LDL-cholesterol (Fig. 1(b), Table 1), we observed a comparable pattern. After a small decrease immediately following the meal, the concentrations reached their lowest point at 2 h with a decrease of −0·1 mmol/l (−4·3 %). Subsequently, the values increased and peaked at 24 h, with an increase of +0·17 mmol/l (+6·9 %). For the TAG (Fig. 1(c), Table 1), we observed fluctuations in concentrations over time. Following the meal, there was an initial increase in TAG concentrations, which peaked at 3 h with an increase of 0·25 mmol/l (+27 %). Subsequently, the concentrations appeared to decrease, reaching their lowest values at 10 h with a decrease of −0·13 mmol/l (−17 %). However, the concentrations started to rise again and reached higher levels at 24 h after the meal, showing an increase of +0·13 mmol/l (+13 %) relative to baseline.

We observed that female participants had, on average, consistently higher values of HDL-cholesterol and lower values of LDL-cholesterol and TAG compared with male participants (online Supplementary Table 3). Furthermore, the concentrations of TAG increased more rapidly in male participants after the meal compared with females, although the concentrations peaked at 3 h after the meal for both groups. For HDL-cholesterol and LDL-cholesterol, similar patterns were observed between both sexes (Table 2).

#### Ketone bodies

**Fig. 2.**
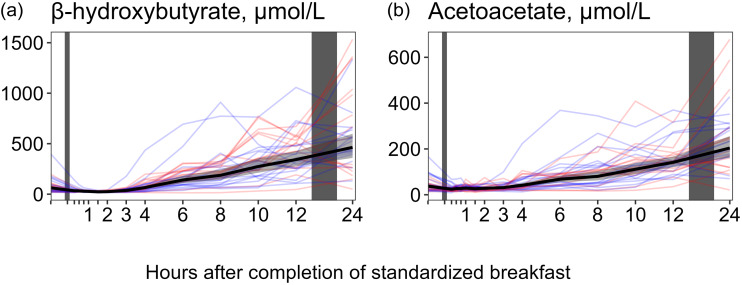
The concentrations of acetoacetate and *β*-hydroxybutyrate as a function of time since completion of the standardised breakfast meal in participants in the Postprandial Metabolism study (*n* 34). The solid black line represents the geometric mean, while the grey-shaded area represents the 95 % geometric CI. The blue and red lines represent the male and female participants, respectively. The leftmost vertical line indicates the time of the standardised breakfast meal, while the rightmost vertical line indicates time spent outside the study centre.

For both AcAc and *β*HB (Fig. 2, Table 1), we observed a slight decrease in concentrations shortly after the meal. For AcAc, the decrease was −35·5 % at 15 min, while for *ß*HB, it was −63·1 % at 90 min. However, starting from approximately 3 h after the meal, the concentrations of both metabolites began to steadily increase. The highest values were observed at 24 h, with an average increase of +433 % for AcAc and +633 % for *ß*HB. A similar pattern was observed between both sexes (Table 2).

#### Acylcarnitines

**Fig. 3.**
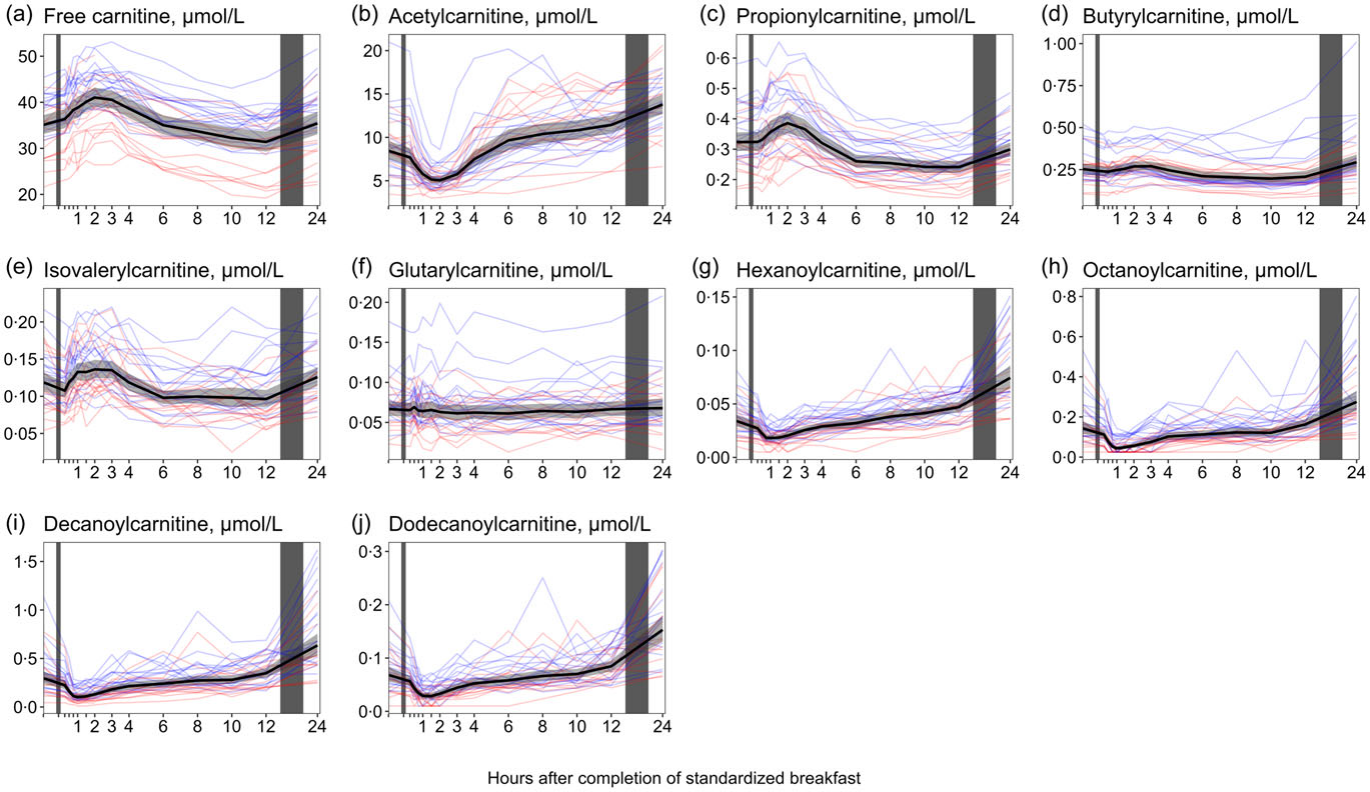
The concentrations of free carnitine and acylcarnitines as a function of time since completion of the standardised breakfast meal in participants in the Postprandial Metabolism study (*n* 34). The solid black line represents the geometric mean, while the grey-shaded area represents the 95 % geometric CI. The blue and red lines represent the male and female participants, respectively. The leftmost vertical line indicates the time of the standardised breakfast meal, while the rightmost vertical line indicates time spent outside the study centre.

For free carnitine (Fig. 3(a), Table 1), we observed an immediate increase in concentrations after the meal, reaching the highest values at 2 h with a + 17 % increase. Subsequently, the concentrations gradually decreased until 12 h (−10 %) before returning to baseline values at 24 h. We found that females had, on average, consistently lower concentrations of free carnitine than males at all timepoints (online Supplementary Table 3). However, we observed no considerable differences between the sexes in terms of the relative change after the meal (Table 2).

Regarding the short-chain acylcarnitines (C2−C5, Fig. 3(b)–(f), Table 1), we observed a decrease in acetylcarnitine (C2) concentrations after the meal, reaching the lowest values at 2 h (−40 %), followed by a steady increase until reaching the highest values at 24 h (+63 %). For propionylcarnitine (C3) and isovalerylcarnitine (iC5), concentrations increased immediately after the meal, reaching the highest values at 2 h (+19·2 % and 14·6 % increase, respectively) before returning to baseline values. Thereafter, concentrations decreased and remained lowered from 6 to 12 h, before values slightly increased at 24 h. Similar observations were observed for butyrylcarnitine (C4), with concentrations decreasing from 4 h after the meal, reaching the lowest values at 10 h (−22 % decrease) and subsequently increasing until 24 h after the meal (+17 % increase). The concentrations of glutarylcarnitine (C5-dicarboxyl) showed minimal changes in response to dietary intake and fasting. Female participants had, on average, consistently lower concentrations of C3, C4, iC5 and C5-dicarboxyl at all timepoints (Supplementary Table 3), while the relative changes appeared similar between sexes (Table 2).

For the medium-chain acylcarnitines (hexanoylcarnitine [C6], octanoylcarnitine [C8], decanoylcarnitine [C10] and dodecanoylcarnitine [C12], Fig. 3(g)–(j), Table 1), we observed a common pattern with decreased concentrations immediately after the meal. All these acylcarnitines reached their lowest levels at 60 min, with decreases ranging from −47 % (C6) to −70 % (C8). Subsequently, concentrations steadily increased, reaching the highest values at 24 h, with increases ranging from +86 % (C8) to +120 % (C12). Female participants tended to have lower concentrations of all medium-chain acylcarnitines compared with male participants (online Supplementary Table 3); however, the patterns in concentrations after the meal were similar between both sexes (Table 2).

## Discussion

In the present study, including eighteen males and sixteen females, we investigated biomarker concentrations at thirteen timepoints after a standardised meal, until 24 h after the meal. We observed that the concentrations of HDL-cholesterol and LDL-cholesterol decreased, while TAG concentrations increased during the first hours after the meal. The concentrations of AcAc and *ß*HB, acetylcarnitine and the medium-chain acylcarnitines decreased immediately after the meal but thereafter increased and reached the highest values at 24 h. Free carnitine, propionylcarnitine, butyrylcarnitine and isovalerylcarnitine peaked during the first hours after the meal before returning to baseline values and thereafter increasing until 24 h.

### Main findings

For the lipids, the slight decrease in HDL-cholesterol and LDL-cholesterol serum concentrations shortly after the meal aligns with previous findings reported in the literature^(28–31)^, where a 4–7 % decrease in HDL-cholesterol and LDL-cholesterol has been reported the first hours after habitual dietary intake^(31)^ and a fat-rich meal^(29)^. This response may be explained by augmented cholesterol ester transfer from LDL-cholesterol and HDL-cholesterol particles to very VLDL-cholesterol and chylomicrons, facilitated by cholesteryl ester transfer protein and lipoprotein lipase. This phenomenon is suggested to arise due to an influx of TAG-rich lipoproteins from the intestine^(28,29)^, which stimulates the activity of cholesteryl ester transfer protein and lipoprotein lipase. Additionally, the LDL-cholesterol concentrations may be further decreased due to enhanced TAG hydrolysis in chylomicrons catalysed by lipoprotein lipase after the meal, as this process may inhibit the formation of LDL-cholesterol particles from VLDL-cholesterol because VLDL-cholesterol and chylomicrons compete for lipoprotein lipase^(30,32)^. The geometric mean increase in TAG concentrations of 0·25 mmol/l at 3 h after the meal also aligns with previous findings in the literature where increases of 0·3 mmol/l^(2)^ and 0·2 mmol/l^(33)^ after habitual dietary intake have been reported. The composition of the meal consumed by the participants, containing approximately 20 g of fat, offers a plausible explanation for the evident TAG peak^(32)^.

Our findings for the ketones, free carnitine and medium-chain acylcarnitines, which decreased shortly after the meal and thereafter increased, indicate suppression of *ß*-oxidation of fatty acids after the meal and thereafter increased *ß*-oxidation during fasting, as expected given established knowledge about energy metabolism^(27,34)^. These findings can serve as a compliance measure, indicating that participants adhered to the fasting instructions. Notably, distinctive responses emerged for C3 and iC5 within the acylcarnitine profile. These acylcarnitines are byproducts of branched-chain amino acid degradation, and concentrations increase when branched-chain amino acid metabolism accelerates^(14)^. We have previously demonstrated from this study that branched-chain amino acid concentration peaks immediately after the meal and thereafter progressively rises until 24 h^(20)^, seemingly mirroring the patterns for C3 and iC5.

Overall, the concentration patterns for the lipids, ketones and acylcarnitines appeared similar between the sexes. However, male participants exhibited higher absolute carnitine- and acylcarnitine concentrations than females – a finding consistent with previous literature^(17–19)^. It has been suggested that oestrogen decreases serum carnitine concentrations, although the exact mechanisms are not known^(18,19)^. Furthermore, prior studies have shown that oral contraceptives lower total serum carnitine levels in females^(35)^, potentially explaining the sex-based distinctions noted within our study. In addition, male participants had, on average, higher levels of TAG and LDL-cholesterol and lower levels of HDL-cholesterol than female participants, which is also in line with previous literature. It is thought that this is caused by differences in the sex hormones, while it has also been argued that other factors, possibly sex-specific, must be involved. However, the exact nature of these other factors is unclear^(36)^.

### Implications

Our study results shed light on important implications concerning blood lipids and acylcarnitines. We observed that the blood lipids fluctuated after the meal, and while a 2016 consensus statement by the European Atherosclerosis Society and the European Federation of Clinical Chemistry and Laboratory Medicine classified changes in this order of magnitude as clinically insignificant^(4)^, the importance in clinical practice should not be overlooked. Given that predefined cutoffs inform diagnoses and treatment initiations, even slight lipid concentration changes based on the time of blood sampling relative to the last meal could influence disease classification and treatment decisions. For instance, distinct cutoffs for TAG levels are used to classify individuals with normal (< 1·7 mmol/l), moderate (1·7–5·6 mmol/l), moderate to severe (5·65–11·3 mmol/l) and severe (> 11·3 mmol/l) hypertriglyceridaemia^(37)^. As TAG levels change in the hours after dietary intake and during fasting, there is a possibility that some individuals may cross one of the given cutoffs and thus be put in a category based on the timing of the blood sample. Thus, at the very least, it is relevant to consider the time since the last meal when evaluating the lipid profile. This could be done by developing and applying correction factors to the measured concentrations to estimate the concentrations at a timepoint consistent with the cutoffs. Another option is establishing different diagnostic cutoff values for lipid concentrations based on the timing of blood sample collection, potentially improving the sensitivity and specificity when used as diagnostic biomarkers. Furthermore, when the lipids are used as monitoring biomarkers to evaluate the effect of an intervention, such as a lifestyle change or a medical treatment, blood samples for the repeated lipid measurements should be taken at the same time relative to previous dietary intake. If not, a potential change or a non-change in lipid concentrations, which in reality are due to differences in sampling timepoints, may be wrongfully attributed to other factors, such as the effect or the lack of an effect of an intervention.

Moreover, our findings for the ketone bodies and acylcarnitines demonstrated that their concentrations are influenced by prandial status, exhibiting notable changes within the first 24 h after a meal. Merely distinguishing between fasting and non-fasting samples falls short of capturing this complexity. Currently, many studies exploring the ketone bodies and acylcarnitines have measured these biomarkers either after overnight fasting^(8,11,38–42)^ or distinguished between fasting and non-fasting blood samples^(7)^ or not considered the timing of the last meal at all^(12,43)^. Our findings suggest that not considering the timing of the last dietary intake for ketone bodies and acylcarnitine profiling in epidemiological studies could hamper accurate result interpretation and potentially lead to the wrong conclusions of the study. If our findings are confirmed in future studies, we recommend rigorous adherence to standardised sampling procedures that account for the specific duration since the last meal. If standardisation is not possible, the time since the last meal at the time of blood sampling should be recorded and accounted for.

Moving forward, future research should investigate the dynamics of lipid, ketone and acylcarnitine concentrations in more diverse populations, encompassing a broader range of ages, BMI categories and different ethnicities. Moreover, future studies should seek to investigate the dynamics of biomarker concentrations after other meals and during a day with several consecutive meals, mimicking a real-world setting. By addressing these areas, future research could contribute to a more comprehensive understanding of how lipids, ketones and acylcarnitines change and provide the basis for improving the utilisation of these as biomarkers.

### Strength and limitations

Several strengths of this study merit attention. First, we obtained data on a homogenous group of participants, reducing potential variability in metabolite concentrations linked to age, health status and body composition. Unfortunately, the homogeneity of the cohort reduces the generalizability of our findings, and future studies should seek to investigate biomarker and metabolite concentrations in cohorts of other ages, ethnicities and health statuses. Moreover, despite the homogeneity of the cohort, there were large interindividual differences in resting metabolic rate (ranging from 1053 to 2200 kcal/d) and body composition (fat mass percentage ranging from 10·5 to 39·1 among males and 21·6 to 41·2 among females) between the participants^(20)^. It is well-known that body composition may affect postprandial responses^(44)^. As the same meal was provided to all participants, it is likely that the meal was metabolised at different rates, which introduces a source of variability in the results. It should also be underscored that the results are only in the context of a ∼500 kcal meal. The results from this study do not necessarily apply to meals of other sizes and compositions. We did not account for the use of contraceptives or the menstrual cycle phase for female participants, which is a potential source of variability in the results. To minimise interindividual differences in the metabolome before the study visit, all participants were given instructions on physical activity, alcohol consumption and smoking the prior day before the study visit. They were also instructed to consume a semi-standardised meal 12 h before attendance, as recommended by LaBarre and colleagues^(1)^. However, the participants´ carnitine intake was not monitored in the last days before the study visit, which is considered a limitation^(17)^. To reduce the potential impact of circadian rhythm on metabolite concentrations^(45)^, the data collection was conducted at the same time of day for all participants. Participants were outside of the study centre between the 12 h and 24 h blood samples, and it remains possible that they consumed food during this period. Nevertheless, all participants reported adherence to the study protocol during the 24 h period, a claim supported by the observed levels of the ketone bodies. Also, due to the absence of blood samples from 12 to 24 h, we have not captured the dynamics of biomarker concentrations during the fasting state, which is considered a limitation.

### Conclusion

HDL-cholesterol and LDL-cholesterol concentrations decreased, while TAG concentrations peaked within the first 3 h after the consumption of a standardised meal containing ∼500 kcal. The ketone bodies, acetylcarnitine and the medium-chain acylcarnitines decreased the first hours after the meal and thereafter increased, reaching the highest values at 24 h. Free carnitine, propionylcarnitine and isovalerylcarnitine peaked 2 h after the meal before decreasing until 12 h and thereafter returning to baseline values. Our findings suggest that merely distinguishing between fasting and non-fasting blood samples falls short of capturing the dynamics in postprandial and fasting lipid, ketone, carnitine and acylcarnitine concentrations. To enhance the utility of serum ketone bodies, carnitine and acylcarnitine analyses, we recommend to consider accounting for the specific time since the last meal at the time of blood sampling.

## Supporting information

Anfinsen et al. supplementary material Anfinsen et al. supplementary material

## Supplementary material

For supplementary material/s referred to in this article, please visit https://doi.org/10.1017/S0007114524001934.
