# Supplementary material for: Serum concentrations of lipids, ketones and acylcarnitines during the postprandial and fasting state: the Postprandial Metabolism (PoMet) study in healthy young adults
Source: Br J Nutr. 2024 Oct 18;132(7):851–61. doi: 10.1017/S0007114524001934 (PMC11576092; doi:10.1017/S0007114524001934)
Supplement: Anfinsen et al. supplementary material [file S0007114524001934sup001.pdf]

Serum concentrations of lipids, ketones, and acylcarnitines during the postprandial and fasting state: The Postprandial Metabolism in Healthy Young Adults (PoMet) study.
Åslaug Matre Anfinsen.

Supplementary Table 1. Baseline concentrations of serum ketones, lipids, and acylcarnitines measured in the Postprandial Metabolism Study[1]

Biomarker	gMean (gSD)	Mean (SD)	Minimum	Maximum
**Ketones**				
Acetoacetate, µmol/L	37.4 (1.88)	45.9 (33.1)	13.1	167
ß-hydroxybutyrate, µmol/L	62.6 (2.12)	83.2 (73.9)	17.3	399
**Lipids**				
HDL-C, mmol/L	1.55 (1.24)	1.58 (0.36)	1.0	2.8
LDL-C, mmol/L	2.51 (1.31)	2.61 (0.72)	1.6	4.3
Triglycerides, mmol/L	0.85 (1.46)	0.92 (0.36)	0.5	1.82
**Carnitines**				
Free carnitine, µmol/L	35.1 (1.19)	35.6 (5.77)	21.6	45.4
Short-chain acylcarnitines				
Acetylcarnitine (C2), µmol/L	8.45 (1.45)	9.03 (3.43)	3.45	21
Propionylcarnitine (C3), µmol/L	0.32 (1.32)	0.34 (0.09)	0.17	0.58
Butyrylcarnitine (C4), µmol/L	0.25 (1.46)	0.27 (0.10)	0.11	0.52
Isovalerylcarnitine (iC5), µmol/L	0.12 (1.37)	0.12 (0.04)	0.05	0.217
Glutarylcarnitine (C5-DC), µmol/L	0.07 (1.49)	0.07 (0.03)	0.02	0.18
Medium-chain acylcarnitines				
Hexanoylcarnitine (C6), µmol/L	0.03 (1.61)	0.04 (0.02)	0.01	0.08
Octanoylcarnitine (C8), µmol/L	0.14 (1.88)	0.17 (0.11)	0.03	0.53
Decanoylcarnitine (C10), µmol/L	0.30 (1.89)	0.36 (0.22)	0.04	1.14
Dodecanoylcarnitine (C12), µmol/L	0.07 (1.82)	0.08 (0.04)	0.01	0.21

[1]The ketone bodies were analyzed using gas chromatography-tandem mass spectrometry (GC-MS/MS), while the carnitines were analyzed using liquid chromatography-tandem mass spectrometry (LC-MS/MS) at Bevital AS (https://bevital.no/). The lipids were analyzed at the Department of Medical Biochemistry and Pharmacology, Haukeland University Hospital using photometry.

Abbreviations: gMean, geometric mean; gSD, geometric standard deviation; HDL-C, High-density lipoprotein cholesterol; LDL-C, Low-density lipoprotein cholesterol.

Serum concentrations of lipids, ketones, and acylcarnitines during the postprandial and fasting state: The Postprandial Metabolism in Healthy Young Adults (PoMet) study.
Åslaug Matre Anfinsen.

Supplementary Table 2. Serum metabolite concentrations after the consumption of a standardized meal in healthy subjects in the Postprandial Metabolism Study[1]

	Time after meal													
**Biomarker**	**Ref**[2]	**15 min**	**30 min**	**45 min**	**60 min**	**90 min**	**2 hours**	**3 hours**	**4 hours**	**6 hours**	**8 hours**	**10 hours**	**12 hours**	**24 hours**
n	34	34	34	34	34	34	34	33	33	33	33	33	33	33
**Ketones**														
Acetoacetate, µmol/L	37.4	24.1	26.8	27.2	29.2	26.2	28.2	31.3	41.5	67.8	80.4	111	141	204
ß -hydroxybutyrate, µmol/L	62.6	33.8	32.3	29.3	27.4	23.1	24.9	36.6	65.6	141	187	274	343	464
**Lipids**														
HDL-C, mmol/L	1.55	1.53	1.49	1.49	1.49	1.48	1.49	1.50	1.50	1.55	1.58	1.61	1.64	1.56
LDL-C, mmol/L	2.51	2.47	2.42	2.42	2.43	2.42	2.41	2.44	2.46	2.53	2.59	2.65	2.67	2.68
Triglycerides, mmol/L	0.85	0.86	0.91	0.94	0.95	0.99	1.02	1.10	1.09	0.85	0.74	0.72	0.74	0.98
**Carnitines**														
Free carnitine, µmol/L	35.1	36.4	37.3	38.3	38.8	40.1	41.0	40.5	38.6	35.0	33.7	32.3	31.4	35.4
Short-chain acylcarnitines														
Acetylcarnitine (C2), µmol/L	8.45	7.70	6.96	6.33	5.78	5.14	5.05	5.75	7.47	9.65	10.4	10.8	11.4	13.8
Propionylcarnitine, (C3), µmol/L	0.32	0.32	0.33	0.34	0.36	0.37	0.39	0.37	0.32	0.26	0.25	0.24	0.24	0.30
Butyrylcarnitine (C4), µmol/L	0.25	0.24	0.24	0.24	0.25	0.26	0.27	0.27	0.25	0.21	0.20	0.20	0.21	0.29

Serum concentrations of lipids, ketones, and acylcarnitines during the postprandial and fasting state: The Postprandial Metabolism in Healthy Young Adults (PoMet) study.
Åslaug Matre Anfinsen.

Biomarker	Time after meal													
	**Ref[2]**	**15 min**	**30 min**	**45 min**	**60 min**	**90 min**	**2 hours**	**3 hours**	**4 hours**	**6 hours**	**8 hours**	**10 hours**	**12 hours**	**24 hours**
Isovalerylcarnitine (iC5), µmol/L	0.12	0.11	0.12	0.12	0.13	0.13	0.14	0.14	0.12	0.10	0.10	0.10	0.10	0.13
Glutarylcarnitine (C5-DC), µmol/L	0.07	0.07	0.07	0.06	0.06	0.07	0.06	0.06	0.06	0.06	0.06	0.06	0.07	0.07
Medium-chain acylcarnitines														
Hexanoylcarnitine (C6), µmol/L	0.03	0.03	0.02	0.02	0.02	0.02	0.02	0.03	0.03	0.03	0.04	0.04	0.05	0.07
Octanoylcarnitine (C8), µmol/L	0.14	0.11	0.08	0.05	0.04	0.05	0.06	0.08	0.10	0.11	0.12	0.12	0.16	0.28
Decanoylcarnitine (C10), µmol/L	0.30	0.22	0.16	0.11	0.10	0.11	0.13	0.18	0.21	0.24	0.27	0.28	0.35	0.63
Dodecanoylcarnitine (C12), µmol/L	0.07	0.06	0.04	0.03	0.03	0.03	0.03	0.04	0.05	0.06	0.07	0.07	0.08	0.15

[1]All values are reported as geometric means. [2] Reference values were determined from the pre-breakfast blood draw. **Abbreviations:** HDL-C, High-density lipoprotein cholesterol; LDL-C, Low-density lipoprotein cholesterol

Serum concentrations of lipids, ketones, and acylcarnitines during the postprandial and fasting state: The Postprandial Metabolism in Healthy Young Adults (PoMet) study.

Åslaug Matre Anfinsen.

Supplementary Table 3. Serum metabolite concentrations after the consumption of a standardized meal in male (*n*= 18) and female (*n* = 16) participants in the Postprandial Metabolism Study[1]

Biomarker	Sex[2]	Ref[3]	15 min	30 min	45 min	60 min	90 min	2 hours	3 hours	4 hours	6 hours	8 hours	10 hours	12 hours	24 hours
			**Time after meal**												
n		34	34	34	34	34	34	34	33	33	33	33	33	33	33
**Ketones**															
Acetoacetate, µmol/L	**M**	40.9	27.4	30.4	28.9	30.9	27.9	28.7	32.4	41.2	71.1	83.6	108	148	219
	**F**	33.8	20.9	23.2	25.3	27.4	24.5	27.7	30.0	41.9	64.0	76.7	116	134	187
ß -hydroxybutyrate, µmol/L	**M**	63.8	36.7	33.1	29.3	27.1	23.0	24.5	38.5	63.1	132	183	248	323	479
	**F**	61.2	30.9	31.5	29.3	27.8	23.2	25.5	34.4	68.7	152	192	310	368	445
**Lipids**															
HDL-C, mmol/L	**M**	1.45	1.43	1.39	1.39	1.39	1.40	1.39	1.40	1.41	1.44	1.48	1.51	1.53	1.47
	**F**	1.66	1.64	1.62	1.61	1.62	1.58	1.59	1.62	1.63	1.68	1.73	1.74	1.78	1.69
LDL-C, mmol/L	**M**	2.58	2.55	2.50	2.48	2.50	2.48	2.49	2.52	2.55	2.61	2.65	2.72	2.76	2.80
	**F**	2.44	2.39	2.33	2.35	2.35	2.35	2.32	2.34	2.35	2.44	2.52	2.56	2.57	2.54
Triglycerides, mmol/L	**M**	0.90	0.92	0.95	0.99	1.02	1.08	1.13	1.16	1.16	0.87	0.76	0.73	0.75	1.01
	**F**	0.81	0.81	0.86	0.89	0.87	0.89	0.92	1.02	1.01	0.84	0.72	0.71	0.73	0.95
**Carnitines**															
Free carnitine, µmol/L	**M**	38.3	39.6	40.6	41.5	42.4	43.0	44.2	43.6	41.7	38.3	36.9	35.6	35.1	39.2
	**F**	31.8	33.0	33.9	35.0	35.1	37.1	37.7	37.2	35.3	31.4	30.2	28.6	27.5	31.4
Short-chain acylcarnitines															
Acetylcarnitine (C2), µmol/L	**M**	8.86	8.29	7.50	6.87	6.26	5.48	5.41	6.30	7.53	9.60	10.4	10.6	11.3	14.2
	**F**	8.01	7.08	6.40	5.78	5.29	4.77	4.67	5.15	7.39	9.72	10.3	11.1	11.6	13.3

Serum concentrations of lipids, ketones, and acylcarnitines during the postprandial and fasting state: The Postprandial Metabolism in Healthy Young Adults (PoMet) study.
Åslaug Matre Anfinsen.

Biomarker	Sex[2]	Ref[3]	15 min	30 min	45 min	60 min	90 min	2 hours	3 hours	4 hours	6 hours	8 hours	10 hours	12 hours	24 hours
			**Time after meal**												
n		34	34	34	34	34	34	34	33	33	33	33	33	33	33
Propionylcarnitine (C3), µmol/L	**M**	0.36	0.37	0.38	0.38	0.40	0.42	0.43	0.40	0.35	0.29	0.28	0.27	0.27	0.33
	**F**	0.29	0.28	0.29	0.30	0.31	0.33	0.34	0.33	0.29	0.23	0.22	0.21	0.21	0.27
Butyrylcarnitine (C4), µmol/L	**M**	0.28	0.26	0.26	0.26	0.26	0.28	0.28	0.28	0.26	0.23	0.22	0.21	0.24	0.34
	**F**	0.22	0.22	0.22	0.23	0.23	0.23	0.26	0.26	0.24	0.19	0.18	0.18	0.18	0.25
Isovalerylcarnitine (iC5), µmol/L	**M**	0.13	0.12	0.13	0.14	0.14	0.15	0.15	0.14	0.13	0.11	0.11	0.11	0.12	0.14
	**F**	0.11	0.09	0.11	0.11	0.12	0.12	0.12	0.13	0.11	0.09	0.09	0.09	0.08	0.11
Glutarylcarnitine (C5-DC), µmol/L	**M**	0.07	0.07	0.08	0.07	0.08	0.08	0.07	0.07	0.07	0.07	0.07	0.08	0.07	0.08
	**F**	0.06	0.06	0.06	0.06	0.05	0.05	0.05	0.05	0.05	0.05	0.05	0.05	0.06	0.06
Medium-chain acylcarnitines															
Hexanoylcarnitine (C6), µmol/L	**M**	0.04	0.03	0.03	0.02	0.02	0.02	0.03	0.03	0.03	0.04	0.04	0.04	0.05	0.09
	**F**	0.03	0.02	0.02	0.01	0.01	0.01	0.02	0.02	0.03	0.03	0.03	0.04	0.04	0.06
Octanoylcarnitine (C8), µmol/L	**M**	0.18	0.14	0.11	0.07	0.05	0.07	0.08	0.10	0.13	0.14	0.15	0.15	0.19	0.33
	**F**	0.11	0.08	0.05	0.04	0.03	0.03	0.04	0.05	0.07	0.09	0.09	0.10	0.13	0.22
Decanoylcarnitine (C10), µmol/L	**M**	0.36	0.28	0.21	0.15	0.14	0.16	0.18	0.24	0.27	0.29	0.33	0.33	0.38	0.76
	**F**	0.24	0.18	0.12	0.08	0.07	0.07	0.09	0.13	0.16	0.19	0.22	0.23	0.32	0.51
Dodecanoylcarnitine (C12), µmol/L	**M**	0.08	0.07	0.05	0.04	0.04	0.04	0.04	0.06	0.06	0.07	0.08	0.07	0.09	0.17
	**F**	0.06	0.04	0.03	0.03	0.02	0.02	0.03	0.03	0.04	0.05	0.06	0.07	0.08	0.14

[1]All values are reported as geometric means. [2]Male/Female [3]Reference values were determined from the pre-breakfast blood draw. **Abbreviations:** HDL-C, High-density lipoprotein cholesterol; LDL-C, Low-density lipoprotein cholesterol.

Serum concentrations of lipids, ketones, and acylcarnitines during the postprandial and fasting state: The Postprandial Metabolism in Healthy Young Adults (PoMet) study.
Åslaug Matre Anfinsen.

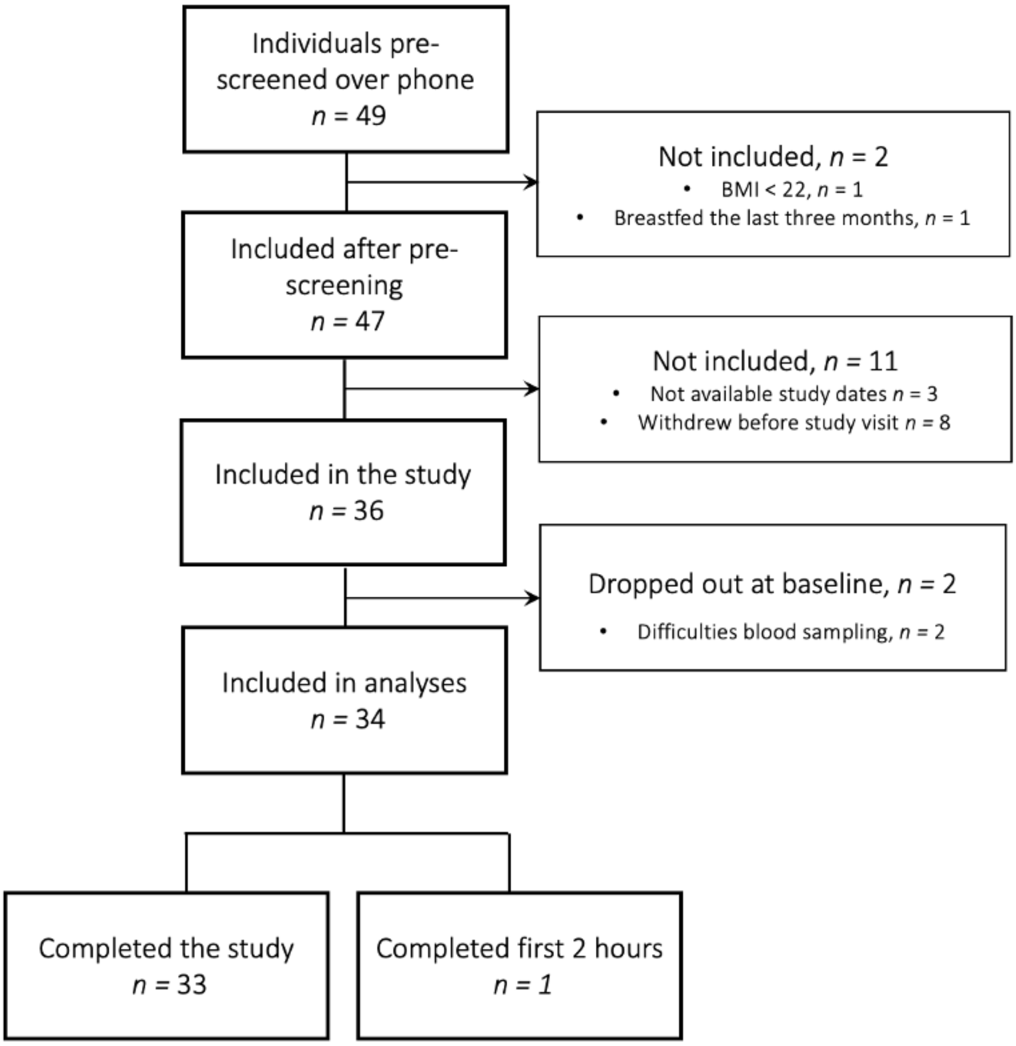

Supplementary Figure 1. Flowchart of the inclusion process for participants in the Postprandial Metabolism Study (Reproduced from Anfinsen *et al.*, 2023, British Journal of Nutrition, doi: 10.1017/S0007114523002490)

Serum concentrations of lipids, ketones, and acylcarnitines during the postprandial and fasting state: The Postprandial Metabolism in Healthy Young Adults (PoMet) study.
Åslaug Matre Anfinsen.

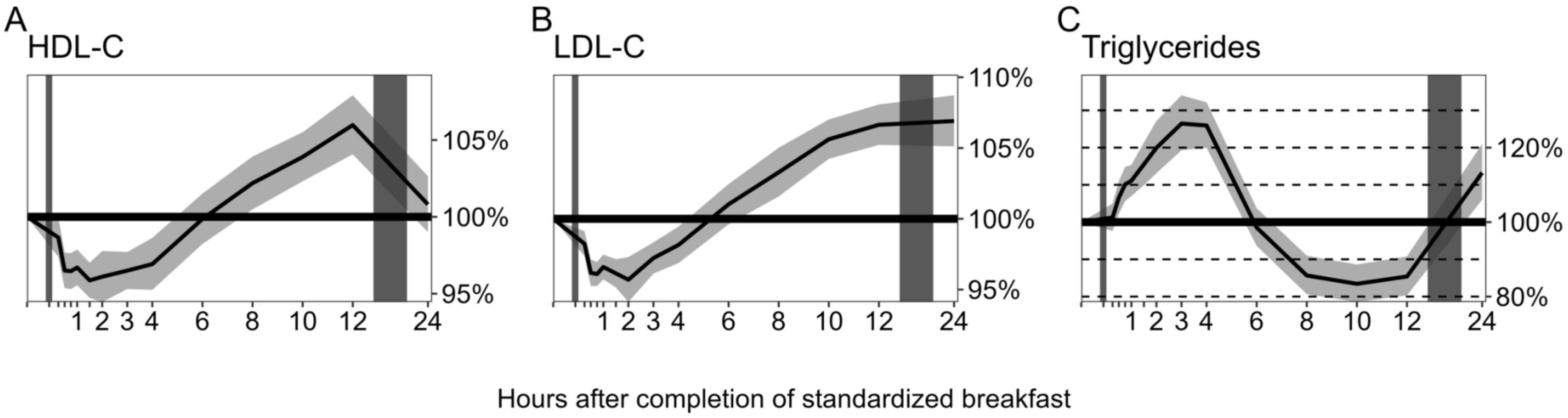

Supplementary Figure 2. The relative change in lipid concentrations (% change from reference values) as a function of time since completion of the standardized breakfast meal in participants in the Postprandial Metabolism Study (n = 34). The solid black line represents the geometric mean, while the grey shaded area represents the 95% geometric confidence intervals. The leftmost vertical line indicates the time of the standardized breakfast meal, while the rightmost vertical line indicates time spent outside the study center.

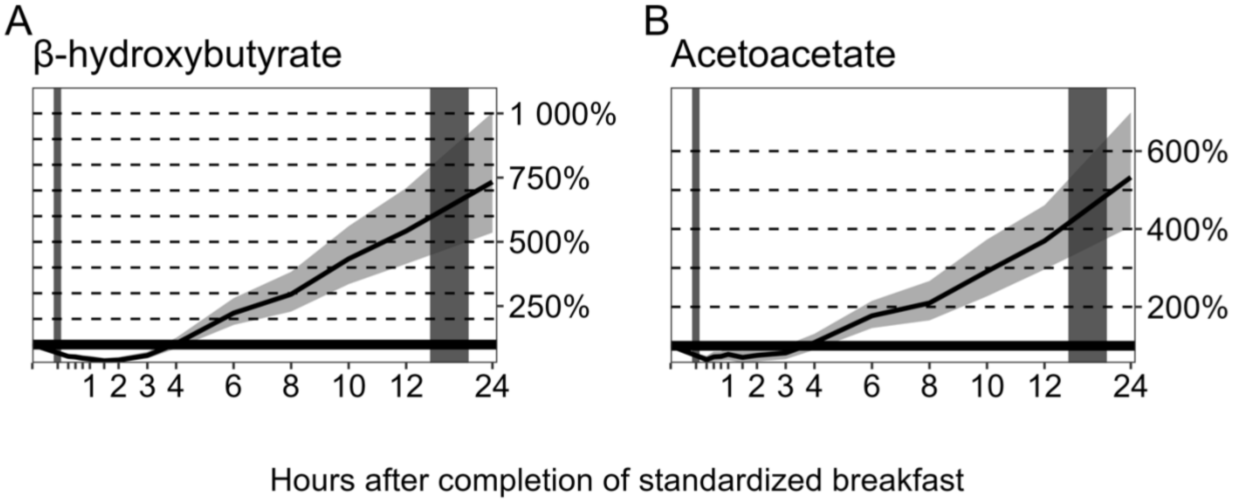

Supplementary Figure 3. The relative change in β-hydroxybutyrate and acetoacetate concentrations (% change from reference values) as a function of time since completion of the standardized breakfast meal in participants in the Postprandial Metabolism Study (*n* = 34). The solid black line represents the geometric mean, while the grey shaded area represents the 95% geometric confidence intervals. The leftmost vertical line indicates the time of the standardized breakfast meal, while the rightmost vertical line indicates time spent outside the study center.

Serum concentrations of lipids, ketones, and acylcarnitines during the postprandial and fasting state: The Postprandial Metabolism in Healthy Young Adults (PoMet) study.
Åslaug Matre Anfinsen.

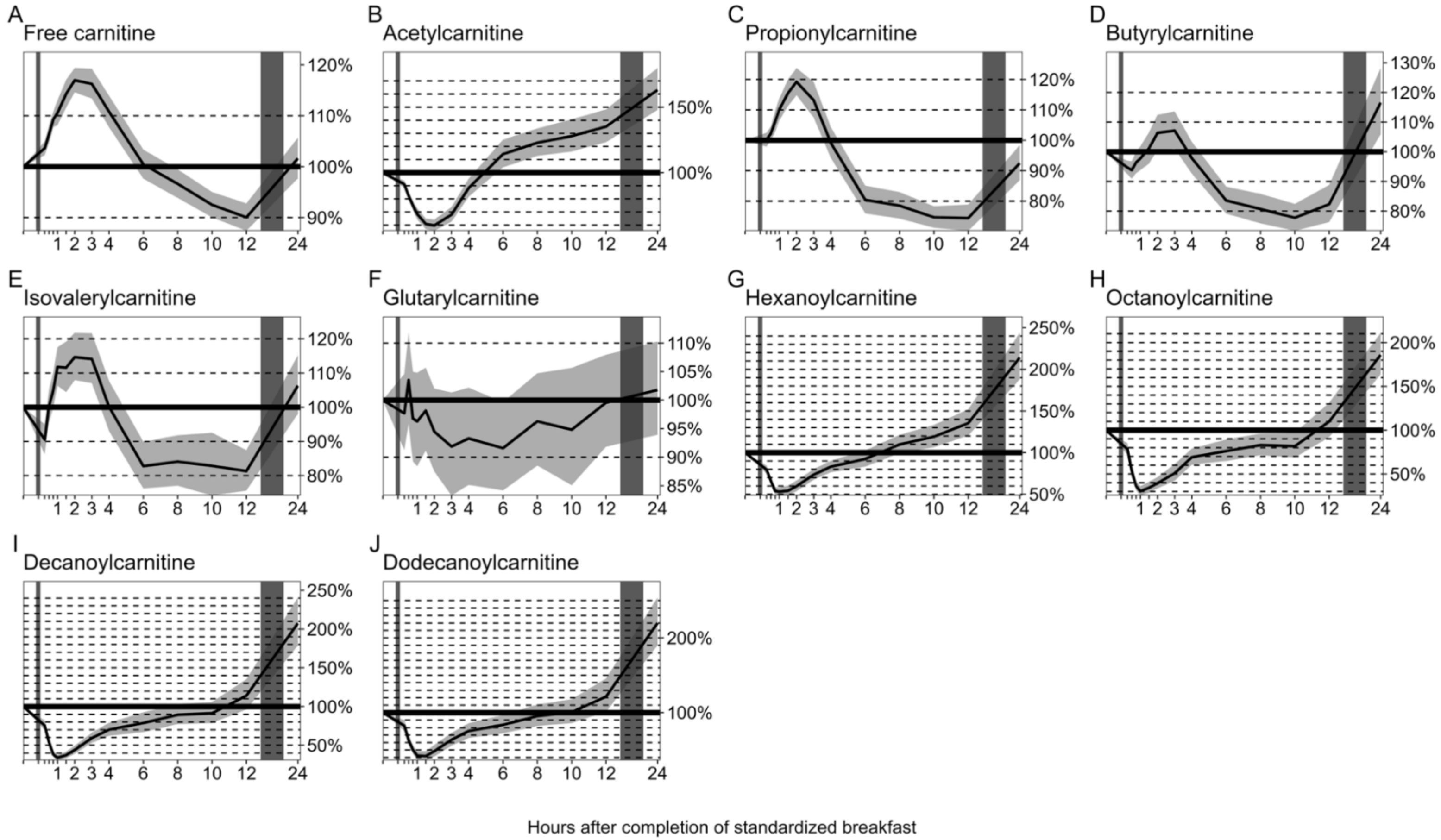

Supplementary Figure 4. The relative change in free carnitine and acylcarnitine concentrations (% change from reference values) as a function of time since completion of the standardized breakfast meal in participants in the Postprandial Metabolism Study (*n* = 34). The solid black line represents the geometric mean, while the grey shaded area represents the 95% geometric confidence intervals. The leftmost vertical line indicates the time of the standardized breakfast meal, while the rightmost vertical line indicates time spent outside the study center.
